# Bangladesh *Chars* Tobacco Assessment Project (CTAP) 2018: a data note

**DOI:** 10.1186/s13104-018-4015-0

**Published:** 2018-12-20

**Authors:** Adnan M. S. Fakir, Mustahsin Aziz, Mutasim Billah Mubde, Afraim Karim, Ashraf S. Khan, Rifayat Raisa, Lubaba Ferdous Alim, Mayeesha Fahmin

**Affiliations:** 1 BRAC University, 66 Mohakhali, Dhaka, 1206 Bangladesh; 2 The World Bank, Plot E 32, Sher-e-Bangla Nagar, Agargaon, Dhaka 1207 Bangladesh; 3 University of Western Australia, 35 Stirling Highway, Crawley, WA 6009 Australia

**Keywords:** Chars Tobacco Assessment Project, CTAP, Bangladesh, South Asia, *Chars*, Smoking, Smokeless, Carbon monoxide, Smokerlyzer, COPD

## Abstract

**Objectives:**

The *Chars* Tobacco Assessment Project 2018 is a holistic survey conducted in the *chars* (riverine islands) of Gaibandha in Northern Bangladesh, covering 985 households over 24 clusters. The survey was conducted with two objectives: (1) to assess levels of tobacco consumption and evaluate prevailing socio-economic, behavioral and health status of the *chars* population, and (2) to look at the effectiveness of advocacy campaigns to reduce tobacco consumption through behavioral nudges via randomized controlled trials (RCTs) in rural Bangladesh. The study site was purposively chosen due to its high tobacco consumption rate, and the geographical segregation of the *chars* aided in reducing spillovers for RCT design.

**Data description:**

In addition to detailed information on tobacco (smoking and smokeless) consumption and perception, data was collected on: household composition, housing and plot ownership, consumption, risks and shocks coping, dowry, farm production, loans, savings and lending, labor income, asset holdings, migration and remittance, anthropometry, respiratory diseases, co-morbidities, reproductive history, risk and time preference. Unique to the dataset are carbon monoxide readings for accurate short term smoking measurement and FEV1 and PEF values for identification of long term lung damage. The data is representative only for the *chars* of Gaibandha.

## Objectives

Bangladesh has one of the highest smoking rates in the world with an age-standardized smoking prevalence of above 34% among men [1]. Poor formulation and execution of tobacco control laws remain one of the prime reasons for such high smoking rates in the country [2]. The situation is further aggravated when the factor of smokeless tobacco (SLT) is taken into consideration. The data of CTAP, 2018, was collected to provide a holistic overview of the socio-economic, behavioral and health conditions of the households in the *chars* of Gaibandha of northern Bangladesh, with a special emphasis on understanding the effect of tobacco (smoking and smokeless) intake on various aspects of their daily lives.

In particular, the baseline dataset was constructed with the following studies in mind:

1. Parental tobacco intake and increased risk of child malnutrition.
2. The effect of smoking on agricultural productivity.
3. Tobacco tug-of-war: anti-tobacco vis-à-vis tobacco sales promotion campaigns.
4. Child pregnancy and its effect on miscarriages and stillbirths.
5. Inter-generational transfer of dowry: can time change a “tradition”?

In addition to the aforementioned studies, the project also ran two randomized controlled trials (RCTs) to assess the effectiveness of two advocacy campaign interventions intended to reduce tobacco consumption of rural households:

6. Repeated nudges through visual warning posters on the primary and secondary harmful effects of tobacco intake;
7. Record keeping of daily tobacco intake to counter non-rational discounting of individual tobacco consumption events.

Smoking status of the participants were assessed though carbon monoxide readings for more accurate measures vis-à-vis self-reported tobacco intake. Both interventions were 4 weeks long when the end-line survey was administered.

## Data description

In addition to detailed information on tobacco (smoking and smokeless) consumption and perception, data was collected on: household composition, housing and plot ownership, consumption, risks and shocks coping, dowry, farm production, loans, savings and lending, labor income, asset holdings, migration and remittance, anthropometry, respiratory diseases, co-morbidities, reproductive history, risk and time preference. Unique to the dataset are carbon monoxide (CO) readings, measured in parts per million (ppm) using the breath smokerlyzer tool, and forced expiratory volume in one second (FEV1) and peak expiratory flow (PEF) values taken using a digital spirometry machine for identification of long-term lung damage. Both tools have been previously used in earlier studies for reliable estimates [3, 4].

The CO readings were taken by asking respondents to exhale into the smokerlyzer after holding their breath for 15 s. This allows a very precise short-term (past 12 h) non-invasive measurement of the level of CO in the bloodstream due to smoking. The ability to have a verifiable measure of the intensity of smoking allows us to overcome the recall-bias inherent in reported measures of daily smoking habits [5]. The FEV1 and PEF values, on the other hand, were taken by asking the respondent to take a deep breath and exhale into the digital spirometry as hard as they could. Respondents were asked to repeat this thrice and the best values were recorded. The values can be referenced against normalized curves by height, weight and age to assess airway obstruction. While there is a growing argument in the available literature advising the use of FEV1 over PEF in measuring bronchial obstruction [6], we leave it to the researcher to decide which one to use for assessment.

### Sampling

The sample for the data collection was selected based on a two-stage clustered sampling approach. In the first stage, 24 *chars* (clusters) from Gaibandha district were randomly selected. The list of 24 *chars* are provided in the questionnaire available in the Harvard Dataverse—see “Data files” section below. At the second stage, from each of the *chars*, 42 households were chosen using a skipping factor of 3 households. After accounting for non-response rates, this sampling approach resulted in a final dataset of 985 households at the baseline. It should be noted that the questionnaire was administered in the local language, Bengali. To ensure minimal discrepancy in translation, the questionnaire was translated and reverse-translated for linguistic consistency checks during pre-testing, which was conducted in a *char* outside of the selected sample.

The sample size and cluster size selected for the study was based on calculations done using the optimal design software. Secondary data collected was been used as a reference for approximating the standardized effect size (equal to 0.318) and in order to discern an effect of 10%, our sampling design provide a power of 83.5% estimated with an intra cluster correlation (ICC) of 0.07, which was obtained from the Global Adult Tobacco Survey (GATS) dataset. End-line data and .do files (for replication purposes) are available upon request after publication of the RCT studies.

### Further studies

The enumerated data was then entered using CSPro and basic consistency checks executed using Stata 14. No additional processing was done to the publicly available dataset and prospective researchers using the dataset to explore particular hypothesis are expected to clean the data and run consistency checks that are specific to the study. As per the set of studies we have conducted using the dataset listed above, under “Objectives” section, our outputs indicate: (1) parental tobacco intake to negatively affect child stunting and underweight measures; (2) smoking to negatively affect agricultural productivity; (3) tobacco sales promotion campaigns to more strongly affect tobacco uptake than anti-tobacco campaigns induce cessation; (4) child marriage to lead to increased probability of miscarriages and stillbirths, and (5) dowry transfers to still be a strong tradition in the *chars* of Gaibandha. Finally, RCT results indicate record keeping of daily tobacco intake to be a significant advocacy strategy to induce tobacco cessation in the short run but impacts of visual nudges remain insignificant. We only succinctly mention the aforementioned findings to aware future researchers of the studies that have already been conducted using this dataset, such that it is easier for them to identify and focus on other gaps in the available literature. The findings are also, of course, open for replication using the data. We believe the dataset has potential for testing many other hypotheses and invite researchers to use the dataset to explore as per their interest.

### Data files

Table 1 provides a technical overview of the dataset which is broken down into 33 separate files in the repository for easier handling.

**Table 1:** Technical overview of data files

Section	Name of data files	File types (file extension)	Data repository and identifier (DOI)
00-1	English Questionnaire	Portable Document Format (.pdf)	Harvard Dataverse (10.7910/dvn/yaav4x) [7]
00-2	Bengali Questionnaire		
00-3	Data codebook		
01	Identification	The following download options are available: Stata 14.0 Binary (.dta); Tab separated values (.tab); RData Format (.RData)	
02	Household composition		
03	Housing		
04	Consumption		
05	Shocks and risks coping		
06a1	HHH’s marriage and dowry information		
06a2	Children’s marriage and dowry information		
06b	Daughter-in-law’s information		
07a	Smoking tobacco consumption of HHH		
07b	Smokeless tobacco consumption of HHH		
07c	Second-hand smoking, and knowledge, attitudes and perception		
08a	Housing and plot ownership		
08b	Farm production		
08c	Farm inputs and labor inputs		
09	Support from government and non-government programs		
10	Loans, savings and lending		
11	Labor income of HHH		
12	Asset holdings		
13	Livestock		
14a	Migration and remittance		
14b	Migration failure		
15	Regular contacts on mainland		
16	Relocation history		
17a	Respiratory diseases		
17b	Co-morbidities and cost of health care		
17c	EQ-5D-3L and WPAI of HHH		
18	Smokeless tobacco consumption of spouse		
19	Labor income of spouse		
20a	EQ-5D-3L and WPAI of spouse		
20b	Reproductive history and antenatal care		
20c	Birth, postnatal care, breastfeeding, and tobacco consumption behaviour during pregnancy		
21a	Risk preference		
21b	Time preference		

## Limitations

The major limitation of the study is that the data is representative only for the *chars* of Gaibandha, hence any findings derived from using this dataset does not hold external validity to a national level or any other dissimilar demography. Furthermore, while the dataset contains details of SLT consumption for females (the spouse of household heads as no household in the sample was female headed), data on smoking tobacco intake was not collected for females due to cultural sensitivity. While the prevalence of such seems to be low in the sample area (based on qualitative findings not reported), this still leaves an avenue open for potential bias while conducting any comprehensive SLT analysis for females. However, if anyone wishes to investigate female vis-a-vis male SLT consumption, linking it other socio-economic factors, that remains to be explored. Finally, it should be mentioned that majority of the data are based on self-reported survey data as opposed to empirical observations (such as the CO, FEV1 and PEF values) and thus may be prone to measurement bias, as in any other field level household survey.

## Abbreviations

CTAP: Chars Tobacco Assessment Project
RCT: randomized controlled trial
FEV1: forced expiratory volume in one second
PEF: peak expiratory flow
CO: carbon monoxide
COPD: chronic obstructive pulmonary disease
ICC: intra cluster correlation
CSPro: Census and Survey Processing System
SLT: smokeless tobacco
